# The Role of Parents' Literacy in Malnutrition of Children Under the Age of Five Years in a Semi-Urban Community of Pakistan: A Case-Control Study

**DOI:** 10.7759/cureus.1316

**Published:** 2017-06-05

**Authors:** Umme K Khattak, Saima P Iqbal, Haider Ghazanfar

**Affiliations:** 1 Community and Family Medicine, Shifa International Hospital, Islamabad, Pakistan; 2 Department of Internal Medicine, Shifa International Hospital, Islamabad, Pakistan

**Keywords:** malnutrition, literacy, child health, community health, public health, prevalence

## Abstract

Objectives: According to a recent survey, Pakistan was ranked as the third highest country with malnutrition and the under-five child mortality. No realistic solution for this growing problem has been found despite the fact that the struggle to tackle the issue of malnutrition among young Pakistani children has been going on for the last several decades. The objective of our study was to look into the relationship between parental education and malnutrition in Pakistan and to make a recommendation to improve the nutritional condition of the children.

Method: We carried a case-control study among 400 mothers from February 2016 to July 2016 in a primary health care center located in a peri-urban community in Pakistan. A self-constructed questionnaire comprising of 75 questions was used to collect the data.

Results: The mean age of mother was found to be 27.61 ± 5.130. The majority of the mothers were uneducated 168 (42.0%) while only 116 (29.0%) fathers were uneducated. About 226 (56.5%) of the children had a normal nutritional status while 102 (25.5%) had first-degree malnutrition, 52 (13.0%) had second-degree malnutrition, and 20 (5.0%) had a third-degree malnutrition. Higher paternal educational status (p = 0.008) and maternal educational status (p = 0.011) were found to be significantly associated with normal child nutritional status.

Conclusion: It is recommended that the education of parents, especially females, in the rural and semi-urban areas should be promoted and given due importance. The focus of all these programs should be the mother in terms of security, employment, literacy, justice, healthcare, food, shelter, and social equality.

## Introduction

Malnutrition is one of the leading causes of childhood mortality worldwide [1]. Deficiency of macronutrients or micronutrients or both can lead to malnutrition. Each year, more than 5.9 million children under the age of five die around the world; 45% of these deaths are attributed to malnutrition [2]. Pakistan is among the countries with the highest prevalence of malnutrition in children under the age of five years. About 44% of the children in Pakistan are stunted, while 31% of the children are underweight [3]. Malnutrition is one of the major causes of death in children under the age of five years in Pakistan [4]. According to United Nations International Children's Emergency Fund (UNICEF), Pakistan had an under-five mortality rate of 81 and ranked 23rd in the world for under-five deaths. Severe malnutrition has been associated with impaired thymic development which results in decreased peripheral lymphocyte count and leads to increases susceptibility to infections [5]. Severe malnutrition also impairs the innate host defense mechanism, further diminishing the immune system of the body. Increased frequency and duration of an infection by itself can lead to malnutrition. This results in a vicious cycle. 

Chronic malnutrition can lead to the development of stunting in children under the age of five years. According to the World Health Organization (WHO), stunting is defined as height-for-age which is more than two standard deviations below the median of WHO child growth standards. According to a meta-analysis, 39.4% of the children living in the regions with the highest prevalence of malnutrition were stunted [6]. Stunting can lead to long-lasting harmful consequences, including decreased cognitive skills, impaired school performance, low productivity, and increased risk of chronic diseases in adult life.

Poverty, food insecurity, and illiteracy are the top three leading causes of malnutrition [7]. A low level of maternal education has been associated with poor feeding practices, leading to malnutrition [8]. Educated mothers are more likely to ensure that their child gets adequate nutrition and treatment. Some studies have found a strong association between maternal education and higher socioeconomic status [9]. Furthermore, uneducated girls have been shown to have a higher probability of being undernourished. Girls who are undernourished have a higher probability of becoming an undernourished mother and, therefore, are at a greater risk of giving birth to low birth weight babies [10]. 

Although extensive studies have been done on maternal education and its impact of malnutrition, there is a dearth of studies done on the impact of paternal education on malnutrition in children under the age of five years. Our study purports to look into the issue of under-five malnutrition through a culturally appropriate lens. For this purpose, we carried a case-control study to look into the relationship of parental education with malnutrition in Pakistan specifically and the developing world in general.

## Materials and methods

We carried a case-control study among 400 mothers from February 2016 to July 2016 in a primary health care center located in a peri-urban community in Pakistan. A peri-urban community is a community located between the city and the countryside and is also known as the rural-urban transition zone. Convenient sampling technique was used to select the participants. We used a self-constructed questionnaire compromising of 75 questions to collect the data. Mothers between the age 18 to 45 years who were a permanent resident of the community for the last six months and had children less than five years living with them were included in this study. Mothers who had moved into the community during the last six months or children with a congenital anomaly or chronic diseases, such as cardiovascular disease, sickle cell disease, tuberculosis, and neural tube defect, were not included in the study.

The key variables of interest include maternal and paternal education, parental occupations, living conditions, family size, and income. Informed consent was taken from all the participants and they were assured that their identity will be kept anonymous. Participants were divided into two groups. One group comprised of mothers who had children with an adequate nutritional status while the other group comprised of mothers who had children who had malnutrition. A total of 250 mothers from each group were approached. Cases and controls were matched for age to avoid any potential bias. Unpaired t-test was used to compare the age of the case group with the control group. There was no significant difference in age between the two group (p > 0.05). The response rate was higher (90.4%) in mothers who had children with an adequate nutritional status as compared to (69.6%) mothers who had children with malnutrition. The overall response rate was 80%. The data from the children was collected by using the anthropometric measures of weight for age, height for age, and mid-arm circumference.

The data was stored and analyzed using the Statistical Package for Social Sciences (SPSS), version 21.0 (IBM SPSS Statistics, Armonk, NY). Descriptive statistics were calculated for quantitative variables. The Mann-Whitney U test was applied to determine the association between the parents' education status and nutritional status. The Mann-Whitney U test was also applied to determine the association between child developmental milestones and nutritional status. A Chi-square test was applied to assess the association between the occupational status of parents and nutritional status of the child. The Chi-square test was applied to assess the association between gender of the child and the nutrition status of the child. Multivariate logistic regression was used to predict the determinant of malnutrition in the child. Variables having a significant association (p < 0.05) were considered as determinant factors for malnutrition in the child.

Ethical approval for the study was granted by the Shifa International Hospital Institutional Review Board and Ethics Committee (approval #373-222-2014).

## Results

The mean age of mother was found to be 27.61 ± 5.13 years. The majority of the mothers were married (394 - 98.5%), while only four (1%) were separated and two (0.5%) were divorced. The majority of the mothers were uneducated (168 - 42.0%), while only 116 (29.0%) fathers were uneducated. Most of the mothers were housewives (370 - 92.5%), while only 30 (7.5%) were working mothers. Only 40 (10%) fathers were unemployed. About 158 (39.5) participants had a joint family system, while 150 (37.5%) participants had a nuclear family system and only 92 (23.0%) participants had an extended family system. Most of the participants (36%) had a family income in the range 5,000 - 10,000 Pakistani Rupees. The median number of family members was eight, while the median number of earning family members was one. The mean age of the children was 2.41 ± 1.17 years. The median number of children born per family was three, while the mean age of the mother at the first childbirth was 20.31 ± 4.03 years. About 228 (57.0%) of the index children were males, while 172 (43.0%) were females. This has been presented in Table 1.

**Table 1:** Demographic Factors

Demographic Variables		Frequency
Marital Status	Married	394 (98.5%)
	Separated	4 (1.0%)
	Divorced	2 (0.5%)
Maternal Education	Uneducated	168 (42.0%)
	Primary	90 (22.5%)
	Secondary	132 (33%)
	Tertiary	10 (2.5%)
Paternal Education	Uneducated	116 (29.0%)
	Primary	82 (20.5%)
	Secondary	170 (42.5%)
	Tertiary	32 (8%)
Maternal Occupation	Housewife	370 (92.5%)
	Working Mother	30 (7.5%)
Paternal Occupation	Unemployed	40 (10%)
	Employed	360 (90%)
Monthly Family Income	< 5000	16 (4.0%)
	5000 - 10,000	144 (36.0%)
	11,000 - 15,000	88 (22.0%)
	16,000 - 20,000	92 (23.0%)
	21,000 - 25,000	30 (7.5%)
	> 25,000	30 (7.5%)
Gender of the Index Child	Male	228 (57.0%)
	Female	172 (43.0%)

The childhood developmental milestones were normal in 312 children (78%) while they were delayed in 88 children (22.0%). About 226 (56.5%) of the children had a normal nutritional status, while 102 (25.5%) had first-degree malnutrition, 52 (13.0%) had second-degree malnutrition, and 20 (5.0%) had a third-degree malnutrition. The mean height of the children was found to be 83.83 ± 11.86 cm, the mean weight was found to be 11.41 ± 2.82 kg, and mean mid-arm circumference was found to be 14.53 ± 1.34cm. 

**Figure 1:**
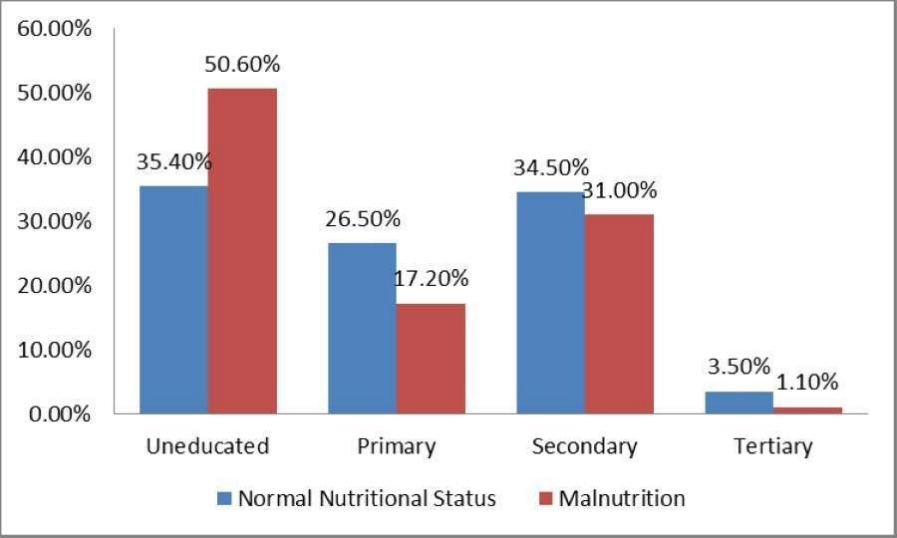
Association of Maternal Educational Status and Nutritional Status

**Figure 2:**
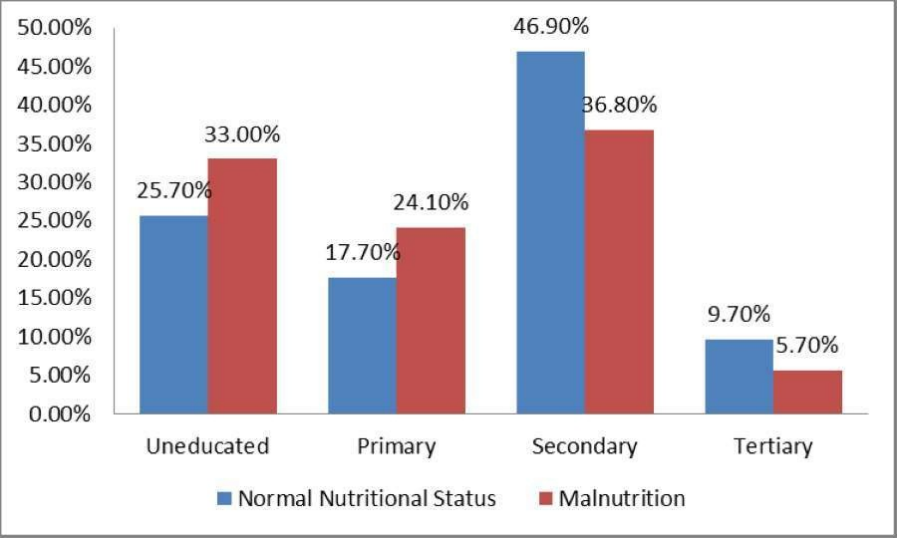
Association of Paternal Educational Status and Nutritional Status

Mann-Whitney U test was applied to determine the association between parents' education status and nutritional status. Higher paternal educational status (p = 0.008) and maternal educational status (p = 0.011) were found to be significantly associated with normal child nutritional status. The impact of parents' educational status on the nutritional status of the children has been shown in Figures 1-2.

The Chi-square test was applied to assess the association between the occupational status of parents and nutritional status of the children. Unemployed mothers were 2.8 times more likely to have children with normal nutritional status as compared to working mothers (p < 0.008), while employed fathers were 2.1 times more likely to have children with normal nutritional status as compared to the unemployed father (p < 0.026). The Chi-square test was also applied to assess the association between the gender and the nutritional status of the children. The male child was 2.23 times more likely to have a normal nutritional status as compared to the female child (p < 0.001) (Table 2).

**Table 2:** Association of Parent’s Occupational Status with Gender and Nutritional Status of the Children

		Normal Nutritional Status	Malnutrition	p-value	Odds Ratio
Maternal Occupation	Unemployed	216	154	0.008	2.8
	Employed	10	20		
		Normal Nutritional Status	Malnutrition	p-value	Odds Ratio
Paternal Occupation	Employed	210	150	0.026	2.1
	Unemployed	16	24		
		Normal Nutritional Status	Malnutrition	p-value	Odds Ratio
Gender of the Children	Male	148	80	<0.001	2.23
	Female	78	94		

In a multivariate analysis, the female child, employed mother, unemployed father, and illiterate mother and father were significantly associated with malnutrition in the children. Children of illiterate mothers were 1.7 times more likely to be malnourished as compared to the children of a literate mother (Table 3).

**Table 3:** Multivariate Analysis of the Determinants of Malnutrition in the Children

Dependent Variable	Adjusted Odds Ratio (AOR)	p-value
Maternal Education Status		
Educated (Ref)		
Illiterate	1.7	0.006
Paternal Education Status		
Educated (Ref)		
Illiterate	1.5	0.003
Maternal Occupational Status		
Unemployed (Ref)		
Employed	3.1	0.004
Paternal Occupational Status		
Employed (Ref)		
Unemployed	2.4	< 0.001
Gender of the Child		
Male (Ref)		
Female	2.7	< 0.001

**Figure 3:**
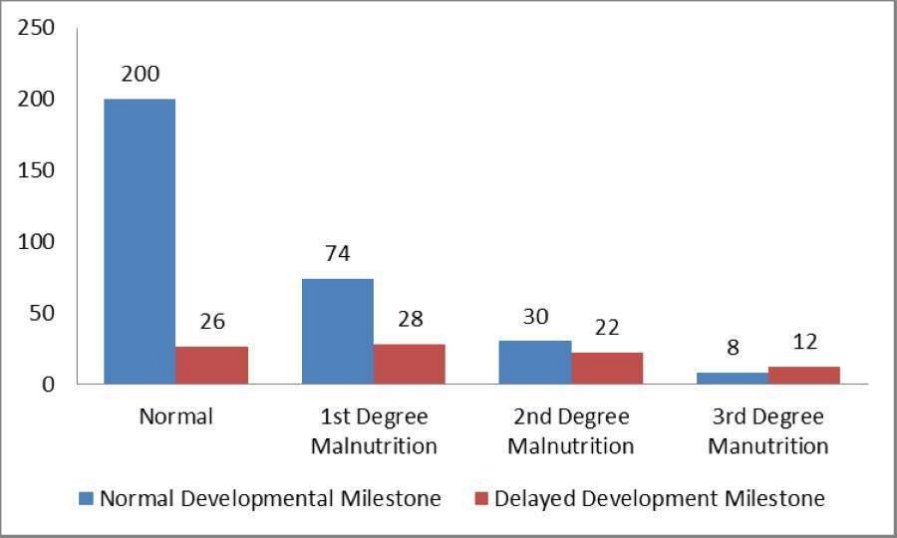
Association of Childhood Developmental Milestone and Nutritional Status

The Mann-Whitney U test was applied to determine the association between the childhood developmental milestones and nutritional status. A higher degree of malnutrition was found to be significantly associated with delayed developmental milestones (p < 0.001) (Figure 3).

## Discussion

Malnutrition is a global issue and presents significant threats to humans. It is the more prevalent public health-related issue in South Asian countries [11]. Malnutrition continues to take its largest toll in Southern Asia, especially in countries like Bangladesh, Pakistan, and India [12]. In our study, the prevalence of malnutrition was found to be 43.5%, which is a little lower than the results of National Nutrition Survey Pakistan 2011, which showed 48% malnutrition. It is also lower than a study done in Karachi, which showed a prevalence of 50% [13]. Poor nutritional status during the early years of life can have a detrimental effect on the semantic memory, learning, concentration, language, and executive control, which are independent of adulthood education achievement [14]. Therefore, it is the need of the hour to take concrete steps to decrease the grave impact of malnutrition on the community.  

Low literacy of parents can result in poor understanding of their children’s heath-related problems and has been found to be associated with malnutrition of children under the age of five years. Uneducated parents are less likely to clearly explain their child’s symptoms to the physician, and this can act as a barrier in their child receiving the best possible care. Uneducated parents won't be able to read and fully understand the health information provided to them in pamphlet forms.

Parents’ education status is one of the most important determinants of malnutrition. Educated parents are more likely to employ better child-care practices as compared to uneducated parents. According to a study done in Bangladesh, children of mothers with secondary or higher education were at a lower risk of childhood stunting (risk ratio (RR): 0.86), underweight (RR: 0.83) and wasting (RR: 0.82) as compared to children of uneducated mothers [15]. Maternal education has been associated with the better nutritional condition during pregnancy and after birth. This has been shown to be an indirect predictor of better child’s health throughout life [16]. A study done in Pakistan concluded that illiterate mothers are more likely to have poor knowledge about the nutritional requirement of their children, which results in unhealthy feeding practices. This is one of the most common reasons of malnutrition among Pakistani children [17-18]. Fathers’ education has been found to be positively associated with child nutrition [19]. Education of the father is also important in most cases as he is the decision maker for the family and his decision can have a significant impact on the health of the children [20]. According to our study, 52.3% children of the uneducated mother were found to have malnutrition, while only 37.1% children of the educated mother were found to have malnutrition. About 50% of children with an uneducated father were found to have malnutrition, while only 40.8% children of the educated father were found to have malnutrition.

According to a study in Nigeria, it was concluded that children of the unemployed mother were more malnourished as compared to children of the employed mother [21]. These results were contrary to results of other studies. A study done in India concluded that 46.15% and 58.97% of children with working mothers were underweight and stunted, respectively. In comparison, only 37.8% and 44.8% of children of unemployed mothers were underweight and stunted, respectively [22]. This might be because of the fact that mothers working outside are less likely to given attention to their children as compared to mothers who are unemployed. In our study, unemployed mothers were 2.8 times more likely to have children with normal nutritional status as compared to working mothers.

## Conclusions

Malnutrition in children is one of the health challenges in Pakistan. Malnutrition-related mortality and morbidity is a burden on national exchequer at one end and on health care institutions on the other. This important issue can be handled with multipronged policies and multidimensional and multisectoral cooperation and integration. The focus of all these programs should be the mother in terms of security, employment, literacy, justice, healthcare, food, shelter, and social equality.
